# Deubiquitinating Enzymes Regulate Skeletal Muscle Mitochondrial Quality Control and Insulin Sensitivity in Patients With Type 2 Diabetes

**DOI:** 10.1002/jcsm.13763

**Published:** 2025-03-04

**Authors:** Wagner S. Dantas, Elizabeth C. Heintz, Elizabeth R. M. Zunica, Jacob T. Mey, Melissa L. Erickson, Kathryn P. Belmont, Analisa L. Taylor, Gangarao Davuluri, Hisashi Fujioka, Ciarán E. Fealy, Charles L. Hoppel, Christopher L. Axelrod, John P. Kirwan

**Affiliations:** ^1^ Integrated Physiology and Molecular Medicine Laboratory, Pennington Biomedical Research Center Louisiana State University Baton Rouge Louisiana USA; ^2^ Lerner Research Institute Cleveland Clinic Foundation Cleveland Ohio USA; ^3^ Cryo‐Electron Microscopy Core Case Western Reserve University Cleveland OH USA; ^4^ School of Medicine Case Western Reserve University Cleveland Ohio USA; ^5^ Center for Mitochondrial Diseases Case Western Reserve University of School of Medicine Cleveland Ohio USA; ^6^ Department of Pharmacology and Medicine Case Western Reserve University Cleveland Ohio USA

**Keywords:** bioenergetics, fission, fusion, mitochondria, obesity, quality control, type 2 diabetes

## Abstract

**Background:**

Activation of mitochondrial fission and quality control occur early in the onset of insulin resistance in human skeletal muscle. We hypothesized that differences in mitochondrial dynamics, structure and bioenergetics in humans would explain the onset and progression of type 2 diabetes (T2D).

**Methods:**

Fifty‐eight sedentary adults (37 ± 12 years) were enrolled into one of three groups: (1) healthy weight (HW), (2) overweight and obesity (Ow/Ob), or (3) T2D. Body composition, aerobic capacity, and insulin sensitivity were assessed during a 3‐day inpatient stay. A fasted skeletal muscle biopsy was obtained to assess mitochondrial functions. C2C12 myoblasts were transfected with FLAG‐HA‐USP15 and FLAG‐HA‐USP30 and harvested to assess mitochondrial dynamics and cellular insulin action.

**Results:**

Insulin sensitivity and aerobic capacity were lower in Ow/Ob (132% and 28%, respectively) and T2D (1024% and 83%, respectively) relative to HW. Patients with T2D presented with elevated skeletal muscle mitochondrial fission (3.2 fold relative to HW and Ow/Ob), decreased fusion, and impairments in quality control. Mitochondrial content was lower in Ow/Ob (26%) and T2D (56%). USP13 (84%), USP15 (96%) and USP30 (53%) expression were increased with decreased Parkin and Ub activation in T2D alone. USP15 (*R* ^2^ = 0.55, *p* < 0.0001) and USP30 (*R* ^2^ = 0.40, *p* < 0.0001) expression negatively correlated with peripheral insulin sensitivity. USP15 and USP30 overexpression activated DRP1 (3.6 and 3.7 fold, respectively) while inhibiting AKT (96% and 81%, respectively) and AS160 (2.1 and 3.5 fold, respectively) phosphorylation.

**Conclusion:**

Mitochondrial fragmentation bypasses defects in mitophagy to sustain skeletal muscle quality control in patients with T2D.

## 1 Introduction

Skeletal muscle mitochondria have long been implicated in the pathogenesis of insulin resistance and T2D. In early reports, it was noted that patients with T2D exhibit depleted skeletal muscle mitochondrial content [1], impaired enzymatic activity [2], and reduced respiratory capacity [3]. However, other laboratories found skeletal muscle oxidative function to be intact or potentially elevated in humans and rodents with T2D [4, 5, 6]. These conflicting observations have spurred exploration of alternative mechanisms whereby mitochondria may mediate the onset and progression of T2D.

Mitochondrial dynamics, or the coordinated events of fission, fusion, repair, and biogenesis, is an evolutionarily conserved process to maintain organelle fitness and preserve inheritance [Reference S1]. Fission is mediated primarily by the cytosolic GTPase dynamin‐related protein 1 (DRP1), which translocates to the outer mitochondrial membrane to initiate scission of tethered mitochondria [7]. The membrane‐bound mitofusin‐1 and mitofusin‐2 (MFN1/MFN2, respectively) coordinate outer mitochondrial membrane fusion. Inner mitochondrial membrane fusion is regulated by optic atrophy‐1 (OPA1), which completes the integration of adjacent mitochondria. Under physiological conditions, the E3‐ubiquitin ligase Parkin is inactive in the cytosol. However, upon loss of mitochondrial membrane potential, (PTEN)‐induced kinase 1 (PINK1) becomes stabilized on the outer mitochondrial membrane [7], and Parkin is recruited from the cytoplasm to the outer mitochondrial membrane, where it is activated through PINK1 phosphorylation. Phosphorylation of ubiquitin by PINK1 is crucial for activating and recruiting Parkin to mitochondria during mitochondrial autophagy (mitophagy). Subsequently, ubiquitination signals lead to the recruitment of p62, an autophagy receptor that acts as a bridge between damaged mitochondria and autophagosomes [Reference S1] [8]. Mitophagy is further controlled by cytosolic and mitochondrial membrane‐bound ubiquitin specific peptidases (USPs), a family of deubiquitinating enzymes (DUBs) that negatively regulate Parkin function [8].

Mitochondrial dynamics has garnered recent attention as a process that may contribute to insulin resistance and T2D [References S2–S5]. In support of this notion, we and others have shown that activation of DRP1‐mediated mitochondrial fission is an early event in the onset of skeletal muscle insulin resistance in humans and rodents [Reference S2] [9]. Furthermore, interventions such as exercise training that alleviate insulin resistance in T2D appear to restore mitochondrial [9, 10] dynamics. However, the specific mechanisms whereby skeletal muscle mitochondrial function contributes to T2D remain largely unknown. We investigated alterations in mitochondrial dynamics across the spectrum of insulin sensitivity and examined their relationship with the morphological and functional characteristics of mitochondria in human skeletal muscle. We hypothesized that patients with T2D would exhibit elevated skeletal muscle DRP1 activity explained by inadequate quality control.

## 2 Methods

### 2.1 Recruitment, Eligibility and Allocation

Sedentary adults, 18–60 years of age, with a body mass index (BMI) between > 18 and < 50 kg/m^2^ were screened for study eligibility. Patients were excluded with evidence of type 1 or 2 diabetes requiring insulin therapy, lack of weight stability (> 5 kg weight change) in the past 6 months, high physical activity (30 min moderate/high‐intensity exercise two or more times weekly), tobacco usage, smokers or individuals who had quit smoking or were using tobacco within the previous 5 years, hypertriglyceridemia (> 400 mg/dL) and/or hypercholesterolemia (> 260 mg/dL), history of significant metabolic, cardiac, cerebrovascular, haematological, pulmonary, gastrointestinal, liver, renal or endocrine disease or cancer that would affect the outcome measures or subject safety, active pregnancy (as evidenced by a positive pregnancy test) or nursing, use of prescription medications with contraindications to study safety, regular use of over the counter medications that could not be discontinued for the study period, history of a partial or complete hysterectomy or contradictions to exercise. Eligible study participants were allocated to one of three groups based on the following criteria: (i) healthy weight (HW): BMI between 18 and 25 kg/m^2^, no prior diagnosis of T2D and no use of glucose‐lowering medications; (ii) overweight/obesity without T2D (Ow/Ob): BMI between 25 and 50 kg/m^2^, no prior diagnosis of T2D and no use of glucose‐lowering medications; and (iii) overweight/obesity with T2D (T2D): BMI between 25 and 50 kg/m^2^ and a prior diagnosis of T2D or meeting one of the American Diabetes Association criteria for T2D (fasting plasma glucose ≥ 126 mg/dL, 2‐h plasma glucose ≥ 200 mg/dL after an oral glucose tolerance test or HbA_1C_ ≥ 6.5%) upon screening. Participants provided written informed consent before participation, and the study was approved by Cleveland Clinic and Pennington Biomedical Research Center Institutional Review Boards. Data from this study were collected between April 2016 and July 2023. A subgroup of patients from this prospective trial participated concomitantly in a trial registered on clinicaltrials.gov (NCT02697201 and NCT02977442, respectively).

### 2.2 Study Design

Following screening, enrolled patients completed a 2‐week weight and diet stabilization period. Then participants were admitted to our Clinical Research Unit for a 3‐day inpatient period during which they were provided standardized meals (55% carbohydrate, 35% fat and 10% protein) based on individual energy needs. On the morning of day 2, participants were assessed for body composition, followed by an incremental aerobic capacity test. They were discharged following completion of testing and readmitted at 1700 h for an additional overnight stay and dietary control period. At 2000 h, patients were placed on NPO status with exception to water for the remainder of study. On the morning of day 3, patients were awoken at 0530 h for catheterization, followed by initiation of a glucose tracer at 0600 h, a muscle biopsy at 0700 h and initiation of the hyperinsulinemic–euglycemic clamp study at 0800 h.

### 2.3 Body Composition

Height and weight were measured in a hospital gown using standard techniques. Dual‐energy X‐ray absorptiometry (DXA, Lunar iDXA; Madison, WI) was used to determine whole body fat and fat‐free mass. Estimation of fat and fat‐free mass content was obtained from iDXA software according to the manufacturer's instructions.

### 2.4 Aerobic Capacity

Maximal oxygen consumption (VO_2max_) was determined using an incremental, graded treadmill exercise test as described previously [11]. Criteria for determination of a maximal test were as follows: (1) oxygen consumption plateau (< 150 mL/min), (2) heart rate within 15 beats of age‐predicted max, (3) respiratory exchange ratio > 1.15 and/or (4) volitional fatigue. Participants were required to achieve three of four criteria in order for the test to be considered maximal.

### 2.5 Biochemical Analyses

Metabolic profiles, including cell counts and lipid concentrations, were analysed on an automated platform as described previously [Reference S6]. Insulin was determined by a commercially available competitive binding radioimmunoassay (Millipore; HI‐14K).

### 2.6 Oral Glucose Tolerance Test

Postprandial glucose tolerance and insulin secretion were determined by an oral glucose tolerance test (OGTT) as described previously [12]. After an overnight fast, patients were seated in a semi‐recumbent position, obtained from an indwelling intravenous line and blood samples collected at baseline (0), 30, 60, 90, 120 and 180 min following the 75‐g glucose challenge samples were. Samples were assayed immediately for glucose (YSI 2300; STAT Plus, Yellow Springs, OH, USA), and plasma was isolated by centrifugation at 1000 rpm for 10 min at 4°C and stored at −80°C until time to assay for insulin and C‐peptide. Calculations for total area of the curve of glucose (tAUC glucose), total area of the curve of insulin (tAUC insulin), total area of the curve of c‐peptide (tAUC c‐peptide), homeostatic model of insulin resistance (HOMA‐IR), disposition index and Matsuda index were made based on equations described previously [13].

### 2.7 Insulin Sensitivity

Insulin sensitivity was determined using a 5‐h, hyperinsulinemic–euglycemic clamp (90 mg/dL, 40 mU·m^−2^·min^−1^), as described previously [11]. Briefly, a primed (3.28 mg/kg) continuous (0.036 mg·kg^−1^·min^−1^) infusion of D‐[6,6‐^2^H_2_]glucose began at −120 min and continued throughout the procedure to calculate hepatic glucose production (HGP). At 0 min, simultaneous infusion of insulin (constant) and 20% dextrose (variable) began. Arterialized heated‐hand venous blood was sampled at 5‐min intervals (YSI 2900 Biochemistry Analyzer; YSI Inc., Yellow Springs, OH), and the glucose infusion rate (GIR) was adjusted in order to maintain plasma glucose at 90 mg/dL. Insulin sensitivity was then calculated as insulin‐stimulated glucose metabolism (M; mg·kg^−1^·min^−1^) divided by plasma insulin (I; μU/mL) over a 30‐min steady‐state period. Plasma for assessing glucose kinetics was deproteinized, extracted, and derivatized before analysis by gas chromatography–mass spectrometry. Isotopic enrichment (mole percent excess) was determined by fitting the fractional abundances (M + 2; *m*/*z* 330)/(M0; *m*/*z* 328) against a calibration curve. The rate of glucose appearance was then derived using the Steele equation [Reference S7]. Patients withheld medications for T2D for at least 48 h prior to the hyperinsulinemic‐euglycemic clamp test. Glucose was evaluated the evening prior to and the morning of the clamp test.

### 2.8 Muscle Biopsy

Skeletal muscle specimens were obtained from the *vastus lateralis* using a modified Bergström biopsy technique at 0800 following a 12‐h overnight fast described previously [Reference S8]. Upon collection, fat and connective tissue samples were dissected and immediately placed into preservation media or frozen in liquid nitrogen. Flash‐frozen muscle samples were then stored at −140°C until the time of analysis.

### 2.9 Western Blot

Muscle homogenates were prepared as described previously [11]. Briefly, muscle tissue was homogenized using a Polytron immersion disperser in ice‐cold Cell Extraction Buffer (Invitrogen) with added protease inhibitor cocktail, 5‐mM phenylmethylsulfonyl fluoride (Sigma), 1‐mM sodium orthovanadate (Sigma) and PhosSTOP (Roche Applied Sciences, Indianapolis, IN). Sample was then loaded onto 4%–20% tris glycine gels (Novex) and separated via sodium dodecyl sulfate polyacrylamide gel electrophoresis at 100 V for 2 h (Invitrogen). The gels were transferred to PVDF membranes and blocked with 5% non‐fat dry milk in tris‐buffered saline with 0.1% Tween‐20 (TBST) for 1 h. Membranes were then incubated overnight with appropriate primary antibodies (see Supplementary Table 1), washed with TBST and incubated with species‐specific horseradish peroxidase‐conjugated secondary antibodies. Immunoreactive proteins were visualized by enhanced chemiluminescence reagent (SuperSignal West Femto, ThermoFisher Scientific) and quantified by densitometric analysis using ImageJ. Phosphorylated proteins were normalized to total protein expression and the loading control (HSC70) unless otherwise stated. Gel‐to‐gel variation and equal protein loading were controlled using a standardized sample on each gel and values were expressed as fold induction relative to healthy weight (HW).

### 2.10 Immunoprecipitation Assay

Muscle homogenates were prepared as described previously [12]. Muscle homogenates were extracted on lysis buffer containing 20 mmol/L Tris (pH 7.4), 137 mmol/L NaCl, 1% NP‐40, 1 mmol/L phenylmethylsulfonyl fluoride, 20% glycerol, 10 mmol/L sodium fluoride, 1 mmol/L sodium orthovanadate, 2 μg/mL leupeptin and aprotinin. The lysates were centrifuged at 12 000 × *g* for 15 min at 4°C. Dynamin‐related protein (DRP1), ubiquitin‐specific peptidase 13 (USP13), ubiquitin‐specific peptidase 15 (USP15) and ubiquitin‐specific peptidase 30 (USP30) antibodies were immunoprecipitated by incubating the lysates with protein A/G agarose beads (Santa Cruz Biotechnology) at 1:500 dilution overnight at 4°C. Protein (200 μg) was probed overnight followed by incubation in horseradish peroxidase–tagged secondary antibody (Veriblot).

### 2.11 Skeletal Muscle Oxidative Phosphorylation (OXPHOS) and Electron Transfer (ET) Capacity

OXPHOS and ET capacity were determined ex vivo from a mitochondrial preparation as described previously [9]. Fresh muscle tissue (10–15 mg) was immediately placed into BIOPS (50‐mM K+‐MES, 20‐mM taurine, 0.5‐mM dithiothreitol, 6.56‐mM MgCl_2_, 5.77‐mM ATP, 15‐mM phosphocreatine, 20‐mM imidazole, pH 7.1, adjusted with 5‐M KOH at 0°C, 10‐mM Ca–EGTA buffer, 2.77‐mM CaK_2_EGTA + 7.23‐mM K_2_EGTA; 0.1‐mM free calcium) solution. The mitochondrial preparations were then transferred to a mitochondrial respiration medium (110‐mM sucrose, 60‐mM K+‐lactobionate, 0.5‐mM EGTA, 3‐mM MgCl_2_, 20‐mM taurine, 10‐mM KH_2_PO_4_, 20‐mM HEPES adjusted to pH 7.1 with KOH at 37°C and 1 g/L de‐fatted BSA). Sample (2–5 mg) was transferred into the Oxygraph chamber containing 2 mL of MiR05, the oxygen content of the chamber was raised to ~450 μM and the background respiration was allowed to stabilize. Oxygen flux was normalized to tissue wet weight [14]. Cytochrome *c* (10 μM) was added to confirm mitochondrial outer membrane integrity.

### 2.12 Mitochondrial Membrane Potential and Network Fragmentation

Mitochondrial membrane potential and morphology were assessed from thin tissue sections and C2C12 myoblasts cells using the fluorophores tetramethylrhodamine (TMRM; Invitrogen) and MitoTracker Deep Red (Invitrogen) as described previously [9]. Selection of the focal plane, image capture, and signal quantification for all studies was performed by a blinded microscopist. Data were analysed independent of acquisition.

### 2.13 Skeletal Muscle Mitochondrial Ultrastructure

Ultrastructural morphology of muscle tissue was examined using transmission electron microscopy (TEM). Briefly, 10–15 mg of muscle tissue was fixed by immersion in a triple aldehyde‐DMSO mixture. Thin sections were stained with acidified uranyl acetate followed by modified Sato's triple lead stain. Mitochondrial density was determined by manual tracing of only clearly discernible outlines of mitochondria on transmission electron micrographs and quantified using threshold analysis in ImageJ. Autophagic vacuoles described the physical features of autophagosomes, which have been defined and described elsewhere [15]. Abnormal mitochondria were identified by their distorted cristae arrangement, matrix dissolution and swollen appearance, as characterized elsewhere [Reference S9]. The counting and identification of autophagic vacuoles followed previously established methods [References S10 and S11], with the total number of autophagosomes normalized per field of view.

### 2.14 Citrate Synthase Activity

Enzymatic activity of citrate synthase was determined in snap‐frozen tissue (~10 mg) using a commercially available colorimetric assay (Sigma‐Aldrich, St. Louis, MO, USA) as described previously [9]. Briefly, tissue was homogenized in 100 μL of ice cold 1× assay buffer and incubated on ice for 10 min. Homogenates were centrifuged at 10 000 × *g* for 5 min at 4°C to pellet tissue debris. The supernatant was transferred to a fresh tube, and protein content was assessed by BCA assay (Thermo Scientific). Ten micrograms of protein lysate suspended in 1× assay buffer containing 30‐mM acetyl CoA and 10‐mM DTNB was plated in duplicate on a 96‐well plate. Absorbance was then measured on a plate reader set to kinetic mode (412 nm, 1.5‐min duration, 10‐s intervals) before and after the addition of 10‐mM oxaloacetate. Data are expressed as μmoL of activity per minute per g protein.

### 2.15 Cell Culture

C2C12 cells (ATCC, Manassas, VA) were cultured with DMEM growth media containing high glucose (25 mM), 10% FBS and 1% penicillin–streptomycin (100 U/mL) in an incubator at 37°C and 5% CO_2_. After reaching ∼80%–90% confluence, myoblasts at passage 4–5 were cultured onto gelatin‐coated 12‐well plates. Stable overexpressed lines of USP15 and USP30 were generated from C2C12 myoblasts by grown cells in proliferation media to about 40%–50% confluence and transfected with Flag‐USP15 (Cat#22570, Addgene) and Flag‐USP30 (Cat#22578, Addgene) using Mirus TransIT‐2020 transfection reagent (Mirus, Madison, WI). Myoblasts were then selected with puromycin 20 μg/L for 24–48 h and then grown to confluence.

To test whether USP15 and USP30 overexpression impairs the insulin signalling of Akt on threonine 308 and AS160 on threonine 642 phosphorylation sites, C2C12 myoblasts were serum starved in low‐glucose DMEM (1 g/L) supplemented with 1% BSA for 3 h, and insulin (100 nmol/L) was added into the wells 30 min before the end of the experiment. Cells were washed two times in ice‐cold PBS with 0.1‐M Na_3_VO_4_, lysed and harvested in ice‐cold Cell Extraction Buffer (Invitrogen) with added protease inhibitor cocktail, 5‐mM phenylmethylsulfonyl fluoride (Sigma), 1‐mM sodium orthovanadate (Sigma) and PhosSTOP (Roche Applied Sciences, Indianapolis, IN). The lysates were purified by centrifugation at 14,000 × *g* for 20 min at 4°C, and protein content was measured using a BCA protein assay kit (Pierce Biotechnology) and assessed by Western blotting. Akt and AS160 samples were run from four independent experiments.

### 2.16 Statistical Analysis and Power

Sample size for the a priori primary outcome (DRP1^Ser 616^ phosphorylation) was estimated using G*Power 3.1. Using our published data [10], we calculated the relationship between insulin sensitivity and DRP1^Ser 616^ phosphorylation, and by extending this calculation to previously derived measures of insulin sensitivity in patients with a HW, Ow/Ob and T2D, we estimated a sample size for a 3 group fixed effects, one way ANOVA with the following input parameters: α error probability = 0.05, power (1 − β error probability) = 0.95 and a standardized effect size (dz) = 0.482. The total combined group sample size was estimated to be 50 patients with a calculated power of 0.96 (F = 3.13). Homoscedasticity was evaluated by Brown‐Forsythe and Bartlett's test. The normality of distribution was assessed visually by a Q‐Q plot and statistically by the Kolmogorov–Smirnov test. Data are presented as mean ± standard error of the mean (SEM) except where otherwise stated. Between‐group differences were assessed by a one‐way analysis of variance followed by Tukey's post hoc test. Associations were calculated by Pearson's correlation coefficient. Generalized linear mixed models were employed to address the influence of baseline covariates on DRP1^Ser 616^ phosphorylation. All statistical analyses were performed using Prism 10 (GraphPad, San Diego, CA) and SAS software v9.1.

## 3 Results

### 3.1 Participant**'**s Characteristics and Biochemical Markers

A total of 2495 individuals were interested in participation, 350 were screened for study eligibility and 78 were eligible for study participation. Twenty eligible participants declined participation, and as such, 58 were assigned to their respective groups (Table S1). No serious adverse events were reported. Patients with T2D were slightly older than the Ow/Ob or HW participants (Table 1). BMI and fat mass were significantly higher in Ow/Ob and T2D (vs. HW). BMI, but not fat mass, was higher in T2D relative to Ow/Ob (Table 1). Absolute and relative oxygen consumption peaks (VO_2max_) were lower in T2D relative to HW and Ow/Ob. Fasting glucose and glycated haemoglobin (HbA_1C_) were significantly higher in T2D relative to Ow/Ob (Table 1). Fasting insulin was significantly elevated in Ow/Ob and T2D groups relative to HW, though it was comparable between Ow/Ob and T2D. However, C‐peptide was significantly greater in T2D than Ow/Ob (Table 1).

**TABLE 1:** Changes in body composition, hemodynamics and biochemical markers in patients with healthy weight (HW), overweight/obesity (Ow/Ob) or overweight/obesity with type 2 diabetes (T2D).

	HW (*n* = 23)	Ow/Ob (*n* = 15)	T2D (*n* = 20)	*p* value		
				HW vs. Ow/Ob	HW vs. T2D	Ow/Ob vs. T2D
Age (years)	28.1 ± 6.9	35.0 ± 7.1	48.2 ± 10.5	0.0419	< 0.0001	< 0.0001
Body composition						
Height (m)	1.70 ± 0.08	1.73 ± 0.09	1.67 ± 0.07	0.6122	0.4171	0.1078
Weight (kg)	65.1 ± 9.4	97.4 ± 13.1	100.4 ± 17.1	< 0.0001	< 0.0001	0.7973
BMI (kg/m^2^)	22.4 ± 1.7	31.9 ± 3.5	35.9 ± 6.5	< 0.0001	< 0.0001	0.0256
Waist (cm)	77.1 ± 8.1	101.8 ± 12.4	112.6 ± 14.1	< 0.0001	< 0.0001	0.0225
Hip (cm)	96.6 ± 6.3	112.1 ± 10.2	119.3 ± 11.8	< 0.0001	< 0.0001	0.0798
Waist to hip ratio	0.79 ± 0.06	0.90 ± 0.08	0.94 ± 0.10	0.0008	< 0.0001	0.3652
Fat mass (%)	28.1 ± 9.8	39.4 ± 7.2	43.3 ± 6.2	0.0003	< 0.0001	0.3406
Android fat (%)	30.1 ± 10.1	50.8 ± 8.0	52.3 ± 6.7	< 0.0001	< 0.0001	0.8758
Gynoid fat (%)	34.2 ± 10.0	40.5 ± 9.2	44.7 ± 8.3	0.1068	0.0015	0.3896
Lean mass (%)	66.9 ± 16.6	57.1 ± 6.4	53.1 ± 6.0	0.0347	0.0008	0.5787
Hemodynamic markers						
SBP (mmHg)	133 ± 14	123 ± 15	119 ± 11	0.0868	0.3301	0.6944
DBP (mmHg)	71 ± 10	79 ± 7	74 ± 6	0.0050	0.3978	0.1170
VO_2max_ (L/min)	2.44 ± 0.8	2.78 ± 0.6	2.01 ± 0.2	0.2824	0.0476	0.0032
VO_2max_ (mL/kg/min)	37.5 ± 9.9	29.4 ± 6.9	20.5 ± 3.4	0.0058	< 0.0001	0.0028
Time to exhaustion (min)	13.7 ± 3.5	12.2 ± 3.0	12.4 ± 3.3	0.4281	0.4630	0.9895
HR peak (bpm)	190.0 ± 11.2	188.3 ± 8.8	163.9 ± 18.3	0.9368	< 0.0001	< 0.0001
RER peak	1.20 ± 0.10	1.19 ± 0.12	1.11 ± 0.08	0.9397	0.0126	0.0615
Biochemical markers						
Fasting glucose (mg/dL)	87.1 ± 5.8	88.2 ± 10.5	154.2 ± 54.6	0.9936	< 0.0001	< 0.0001
Fasting insulin (μU/mL)	6.4 ± 3.0	15.0 ± 8.0	16.3 ± 8.9	0.0013	< 0.0001	0.8454
Fasting C‐peptide (mg/dL)	1.3 ± 0.5	2.5 ± 1.1	4.2 ± 2.1	0.0339	< 0.0001	0.0061
HbA_1C_ (%)	5.1 ± 0.24	5.5 ± 0.38	6.9 ± 0.87	0.1364	< 0.0001	< 0.0001
HbA_1C_ (mmol/mol)	33.1 ± 2.7	37.4 ± 4.2	52.1 ± 9.5	0.1352	< 0.0001	< 0.0001
Cholesterol (mg/dL)	152.6 ± 28.0	180.1 ± 32.4	177.7 ± 32.1	0.0241	0.0311	0.9726
HDL (mg/dL)	52.6 ± 13.4	42.1 ± 7.4	42.6 ± 8.9	0.0129	0.0110	0.9900
LDL (mg/dL)	86.9 ± 26.4	113.0 ± 26.6	100.4 ± 32.6	0.0230	0.2873	0.4109
VLDL (mg/dL)	13.1 ± 5.2	24.9 ± 10.5	29.0 ± 13.6	0.0075	0.0065	0.6808
Triglycerides (mg/dL)	65.0 ± 34.7	125.0 ± 47.1	137.1 ± 79.5	0.0068	0.0003	0.8083
Albumin (g/dL)	3.8 ± 0.3	4.0 ± 0.2	3.7 ± 0.2	0.0770	0.2139	0.0017
Creatinine (mg/dL)	0.72 ± 0.14	0.81 ± 0.16	0.65 ± 0.15	0.2226	0.3022	0.0131
Sodium (mmoL/L)	138.1 ± 2.0	138.6 ± 2.5	136.6 ± 2.2	0.7774	0.0728	0.0295
Total bilirubin (mg/dL)	0.72 ± 0.32	0.64 ± 0.27	0.90 ± 0.60	0.8389	0.3958	0.2146
Alkaline phosphatase (U/L)	47.9 ± 11.3	67.2 ± 9.3	61.1 ± 16.5	0.0002	0.0047	0.3767
AST (U/L)	17.2 ± 4.0	23.6 ± 10.5	27.3 ± 17.8	0.4019	0.0796	0.7031

*Note:* Data are mean ± SD.

Abbreviations: AST, aspartate aminotransferase; BMI, body mass index; DBP, diastolic blood pressure; HbA_1C_, glycated haemoglobin; HDL, high‐density lipoprotein; HR peak, peak heart rate; LDL, low‐density lipoprotein; RER peak, peak respiratory exchange ratio; SBP, systolic blood pressure; VLDL, very‐low‐density lipoprotein; VO_2max_, maximal oxygen consumption.

### 3.2 Obesity and Type 2 Diabetes Are Characterized by Progressive Skeletal Muscle and Hepatic Insulin Resistance

**FIGURE 1:**
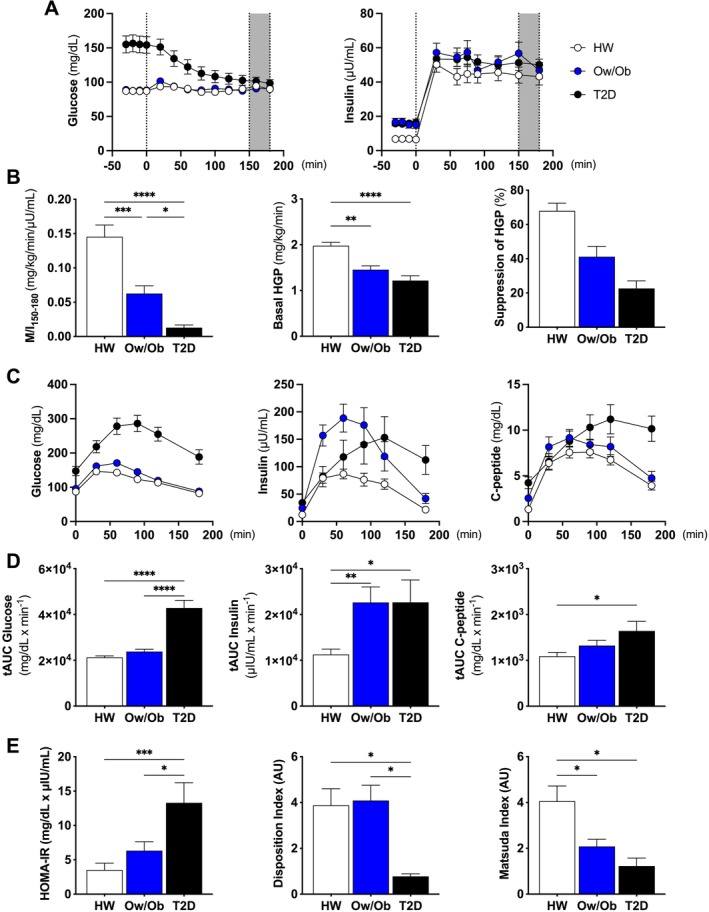
Glucose metabolism, insulin sensitivity and whole‐body substrate metabolism. (A) Fasting glucose and clamp‐derived euglycemia and fasting insulin and clamp‐derived hyperinsulinemia. (B) The ratio of glucose metabolized (M) relative to the prevailing insulin concentration (I) under steady state conditions of the clamp (M/I index), basal hepatic glucose production (HGP) and insulin‐suppressed hepatic glucose production (HGP). (C) Glucose, insulin and C‐peptide concentrations during the oral glucose tolerance test (OGTT). (D) Total area under the curve (tAUC) of glucose, insulin and c‐peptide. (E) Homeostatic model assessment for insulin resistance (HOMA‐IR), disposition index and the Matsuda index. Healthy weight (HW), overweight/obesity (Ow/Ob) and overweight/obesity with type 2 diabetes (T2D). Data are shown as the mean ± SEM. * indicates *p* < 0.05; ** *p* < 0.01; *** *p* < 0.001; **** *p* < 0.0001 for between‐group comparison.

Fasting plasma glucose concentrations were significantly higher in T2D (Figure 1A). In contrast, fasting insulin was higher for both the Ow/Ob and T2D groups prior to the hyperinsulinemic–euglycemic clamp (Figure 1A). Insulin sensitivity based on the insulin‐stimulated rate of glucose disposal (M/I) during the clamp was significantly lower in Ow/Ob and T2D relative to HW and was lowest for the T2D group (Figure 1B). Basal hepatic glucose production (HGP) rate during the clamp was also lower in Ow/Ob and T2D relative to HW (Figure 1B). However, the ability of insulin to suppress HGP was impaired in Ob and was impaired to an even greater extent in T2D (Figure 1B). Responses to the OGTT revealed that glucose tAUC was significantly elevated in T2D compared to HW and Ow/Ob (Figure 1C,D). Insulin tAUC was higher in Ow/Ob and T2D compared to HW, whereas C‐peptide tAUC was elevated in T2D compared to HW (Figure 1D). HOMA‐IR was higher, and the disposition index was lower in T2D compared to both HW and Ow/Ob (Figure 1E). Conversely, the Matsuda index was attenuated in both Ow/Ob and T2D compared to HW (Figure 1E).

### 3.3 Type 2 Diabetes Exacerbates Skeletal Muscle Mitochondrial Fission and Fragmentation

**FIGURE 2:**
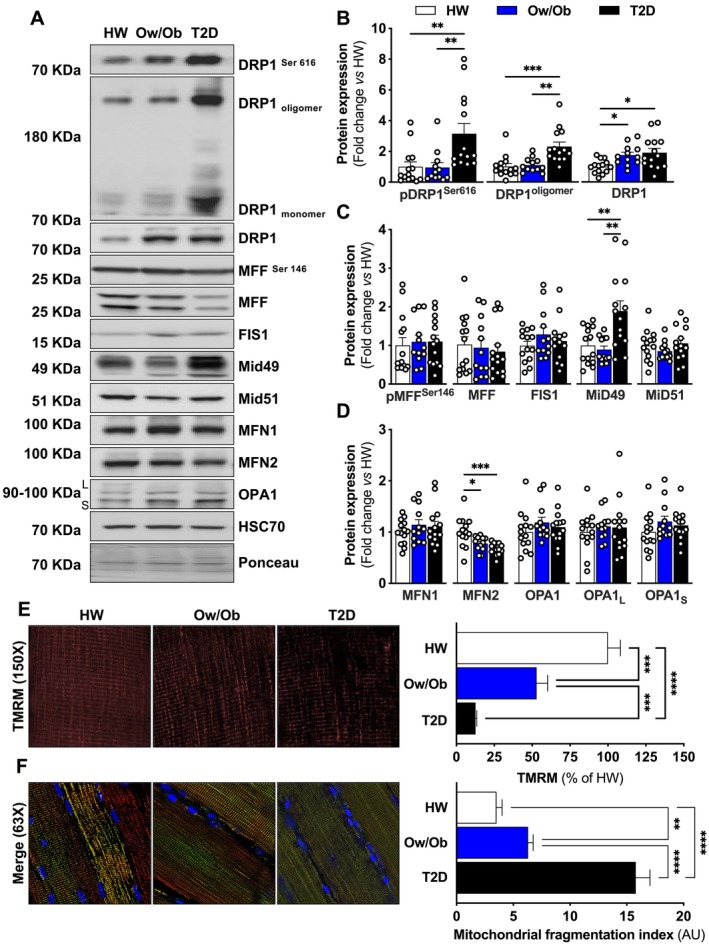
Expression of proteins regulating mitochondrial fission. (A–D) Representative and densitometric quantification immunoblots of phosphorylated and total DRP1, phosphorylated and total MFF, FIS1, Mid49, Mid51, MFN1, MFN2, OPA1 and HSC70 (loading control) relative to healthy weight group (*n* = 14 per group). (E, F). Representative confocal micrographs of resting mitochondrial membrane potential (150× magnification). Micrographs are shown as TMRM alone (E) or (F) merge of TMRM, MitoTracker deep red and Hoechst (63× magnification). (E) Quantification of loss of mitochondrial membrane potential and (F) mitochondrial fragmentation. Healthy weight (HW), overweight/obesity (Ow/Ob) and overweight/obesity with type 2 diabetes (T2D). Data are shown as the mean ± SEM. * indicates *p* < 0.05; ** *p* < 0.01; *** *p* < 0.001; **** *p* < 0.0001 for between‐group comparison.

DRP1 phosphorylation at the Serine 616 site (pDRP1^Ser616^), the primary study outcome, was elevated in T2D compared to Ow/Ob and HW (Figure 2A,B). DRP1 oligomerization, which enables mitochondrial fission by GTP hydrolysis‐mediated DRP1 spiral construction [16], was also higher in T2D compared to Ow/Ob and HW. In contrast, total DRP1 content was greater in both Ow/Ob and T2D (Figure 2A,B). Importantly, adjusting for age, sex and BMI as covariates by linear mixed modelling did not explain the observed group differences in DRP1^Ser 616^ phosphorylation (*R* ^2^ = 0.24, Pr > |t| = 0.8448 HW vs. Ow/Ob; Pr > |t| = 0.2959 HW vs. T2D; Pr > |t| = 0.1899 Ow/Ob vs. T2D). There was no difference in MFF activation at the Serine 146 site (pMFF^Ser146^) between groups, and FIS1 content was also similar for each group (Figure 2A,C). Nevertheless, mitochondrial outer membrane protein 2 (Mid49) content, the protein responsible for recruiting DRP1 to the mitochondrial surface [17], was elevated in T2D relative to Ow/Ob and HW, with no differences in mitochondrial outer membrane protein 1 (Mid51) expression (Figure 2A,C). Related to mitochondrial fusion, MFN2 expression was lower in Ow/Ob and T2D groups compared to HW (Figure 2A,D). However, there were no significant differences in MFN1 and OPA1 expression between these groups (Figure 2A,D). To confirm whether DRP1 hyperactivation led to mitochondrial network fragmentation which would contribute to changes in membrane potential and morphology, we evaluated ex vivo permeabilized muscle fibre bundles stained with tetramethylrhodamine (TMRM) and MitoTracker red. We found that the resting mitochondrial membrane potential progressively declined (Figure 2E,F), while mitochondrial fragmentation increased progressively from HW to T2D (Figures 2E,F and S1). Together, these data suggest that DRP1‐mediated mitochondrial fission may promote skeletal muscle mitochondrial fragmentation in T2D.

### 3.4 Acetylation Does Not Explain DRP1 Hyperactivation in the Skeletal Muscle of Patients With Type 2 Diabetes

DRP1 is primarily regulated by post‐translational modifications such as phosphorylation and acetylation [18]. Indeed, global acetylated‐lysine expression was higher in skeletal muscle of T2D (Figure S2A,B). SIRT1 expression was not different between groups, while SIRT3 and 5 were lower in T2D (Figure S2A,B). Unexpectedly, DRP1 acetylation was not different in T2D skeletal muscle (Figure S2C,D), suggesting there must be another mechanism to explain the hyperactivation of DRP1.

### 3.5 Type 2 Diabetes Differentially Regulates Mitochondrial Content and Fusion

**FIGURE 3:**
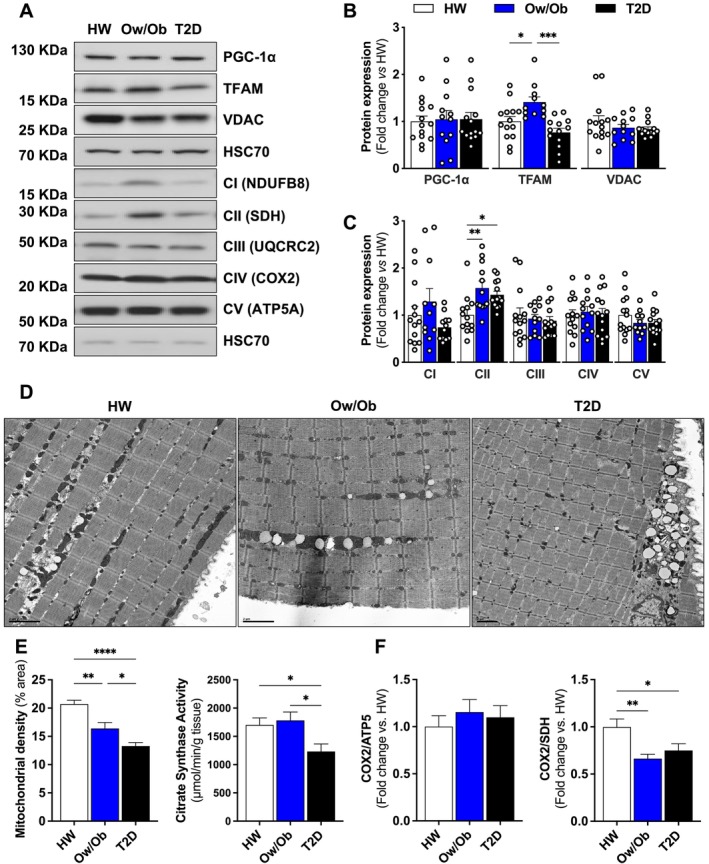
Expression of proteins regulating mitochondrial biogenesis and content. (A–C) Representative immunoblots and densitometric quantification of PGC‐1 α, TFAM, VDAC, respiratory mitochondrial complex I (CI), II (CII), III (CIII), IV, V and HSC70 (loading control) relative to healthy weight group (*n* = 14 per group). (D) Representative images of intermyofibrillar mitochondrial content from transmission electron micrographs (scale bars [black] = 2 μm) (*n* = 3 per group). (E) Quantification of mitochondrial density from the transmission electron micrographs and enzymatic activity of citrate synthase (CS) normalized to protein concentration (*n* = 14 per group). (F) The stoichiometric imbalance ratio between mitochondrial (COX2) and nuclear (ATP5a and SDH) encoded proteins from the respiratory mitochondrial complex. Healthy weight (HW), overweight/obesity (Ow/Ob) and overweight/obesity with type 2 diabetes (T2D). Data are shown as the mean ± SEM. * indicates *p* < 0.05; ** *p* < 0.01; *** *p* < 0.001; **** *p* < 0.0001 for between‐group comparison.

Related to biogenesis, neither the transcriptional coactivator peroxisome proliferator‐activated receptor‐γ coactivator‐1α (PGC‐1α) nor the expression of voltage‐dependent anion channels (VDAC) differed between groups (Figure 3A,B). Although the mitochondrial transcription factor A (TFAM) expression was increased only in Ow/Ob (Figure 3A,B), there were no differences in mitochondrial respiratory complex expression, except for succinate dehydrogenase (SDH) complex II (CII) in the mitochondrial electron transport chain (OXPHOS), which was elevated in Ow/Ob and T2D relative to HW (Figure 3A,C). In contrast, mitochondrial density measured directly by electron microscopy was progressively lower in Ow/Ob and T2D (Figure 3D,E). These data were supported by similar reductions in citrate synthase activity in T2D (Figure 3E). A decline was also observed in the ratio of COX2/SDH in both Ow/Ob and T2D relative to HW (Figure 3F). Collectively, these data suggest that over time, excessive fission in patients with T2D lowers mitochondrial content.

### 3.6 Skeletal Muscle Mitochondrial Proteostasis Signalling Is Impaired in Type 2 Diabetes Despite Intact Oxidative Phosphorylation Capacity

**FIGURE 4:**
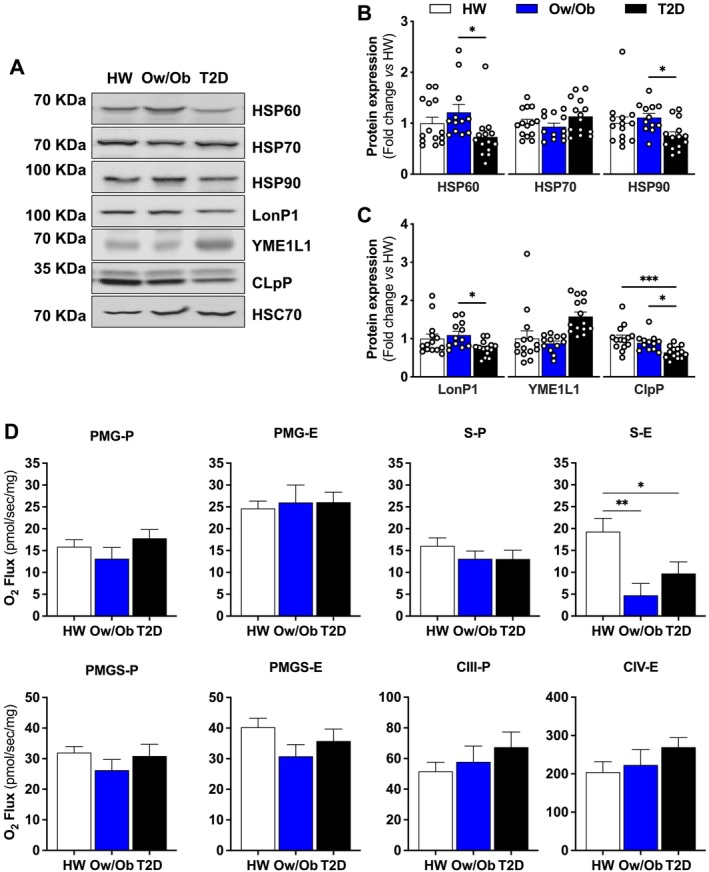
Expression of proteins regulating mitochondrial proteostasis and unfolded protein response and mitochondrial function. (A–C) Representative immunoblots and densitometric quantification of HSP60, HSP70, HSP90, LonP1, YME1L1, CLpP and HSC70 (loading control) relative to healthy weight group (*n* = 14 per group). (D) Assessment of NADH‐linked oxidative phosphorylation (OXPHOS) and electron transfer (ET) capacity, succinate‐linked OXPHOS and ET capacity, convergent NADH‐ and succinate‐linked OXPHOS and ET capacity, complex III OXPHOS and complex IV ET capacity in permeabilized skeletal muscle fibres (*n* = 12–20 per group). Healthy weight (HW), overweight/obesity (Ow/Ob) and overweight/obesity with type 2 diabetes (T2D). Data are shown as the mean ± SEM. * indicates *p* < 0.05; ** *p* < 0.01; *** *p* < 0.001; **** *p* < 0.0001 for between‐group comparison.

Insulin plays a central role in regulating proteome homeostasis (proteostasis) [19]. As a result, hyperglycaemia and hyperinsulinemia negatively impact proteostatic function [20]. To this end, we observed a decline in the mitochondrial retrograde signalling activation in Ow/Ob and T2D groups (Figure 3G,H). Conversely, expression of proteins such as chaperonin (HSP60), mitochondrial heat shock protein 90 (HSP90), mitochondrial Lon peptidase 1 (LonP1) and mitochondrial caseinolytic protease proteolytic subunit (CLpP) were all lower in T2D (Figure 4A–C). However, the expression of mitochondrial ATP‐dependent metalloprotease (YME1L) was exclusively increased in T2D (Figure 4A–C). Impairments in mitochondrial proteostasis signalling were followed by diminished succinate‐linked electron flow in Ow/Ob and T2D (Figure 4G), despite intact NADH‐ and succinate‐linked oxidative phosphorylation and unaltered complex III and IV activity across groups (Figure 4D). Altogether, these data suggest that hyperglycaemia, rather than hyperinsulinemia, leads to loss of mitochondrial proteostasis in skeletal muscle of T2D.

### 3.7 Defective Skeletal Muscle Mitochondrial Quality Control Is a Hallmark of Type 2 Diabetes

**FIGURE 5:**
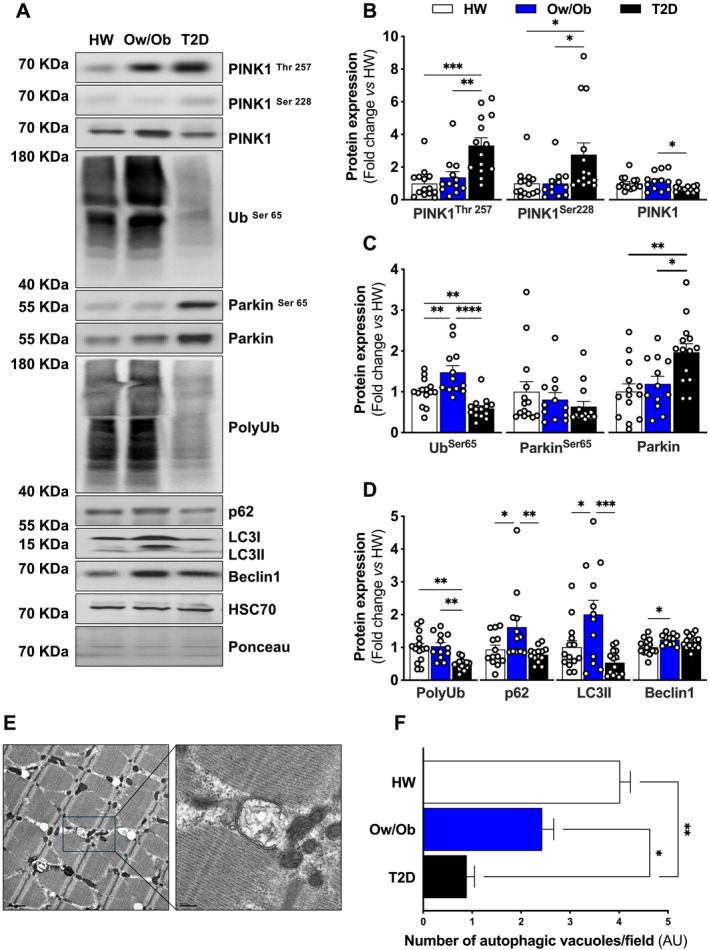
Expression of proteins regulating mitochondrial quality control. (A–D) Representative immunoblots and densitometric quantification of phosphorylated and total PINK1, ubiquitin phosphorylated, phosphorylated and total Parkin, total polyubiquination, p62, LC3II, Beclin1 and HSC70 (loading control) relative to healthy weight group (*n* = 14 per group). (E and F) Representative images and quantification of autophagosomes surrounding mitochondria from transmission electron micrographs (scale bars in black = 0.5 μm) (*n* = 3 per group). Healthy weight (HW), overweight/obesity (Ow/Ob) and overweight/obesity with type 2 diabetes (T2D). Data are shown as the mean ± SEM. * indicates *p* < 0.05; ** *p* < 0.01; *** *p* < 0.001; **** *p* < 0.0001 for between‐group comparison.

We have previously reported that mitochondrial quality control is activated in concert with fission in response to lipid‐induced insulin resistance in humans [9]. Here, we observed that patients with T2D have markedly elevated PINK1 activation at the Threonine 257 (pPINK1^Thr257^) and Serine 228 (pPINK1^Ser228^) sites (Figure 5A,B), whereas ubiquitin (Ub) phosphorylation at the Serine 65 site (pUb^Ser65^) is significantly decreased, and Parkin activation at Serine 65 (pParkin^Ser65^) is unaltered (Figure 5A,C). In line with this observation, polyubiquitination, K63‐linkage polyubiquitin expression, p62 and LC3II expression are lower in T2D, but Beclin1 content is higher for those who have obesity but do not have T2D (Figures 5A,D and S3B). No between‐group differences were observed in free ubiquitin expression (Figure S3A) or K48‐linkage polyubiquitin expression (Figure S3B). Given the evidence of blunted mitochondrial quality control in skeletal muscle of those with T2D, we sought to analyse the number of autophagic vacuoles surrounding mitochondria using electron microscopy micrographs. We observed a reduction in the number of autophagic vacuoles, which provides evidence of impaired mitochondrial quality control in skeletal muscle of T2D (Figure 5E,F). Together, the data suggest that defects in skeletal muscle mitochondrial quality control signalling are fingerprints of T2D.

### 3.8 Deubiquitinases Link the Loss of Mitochondrial Quality Control With Impaired Glucose Uptake in Skeletal Muscle of Patients With Type 2 Diabetes

**FIGURE 6:**
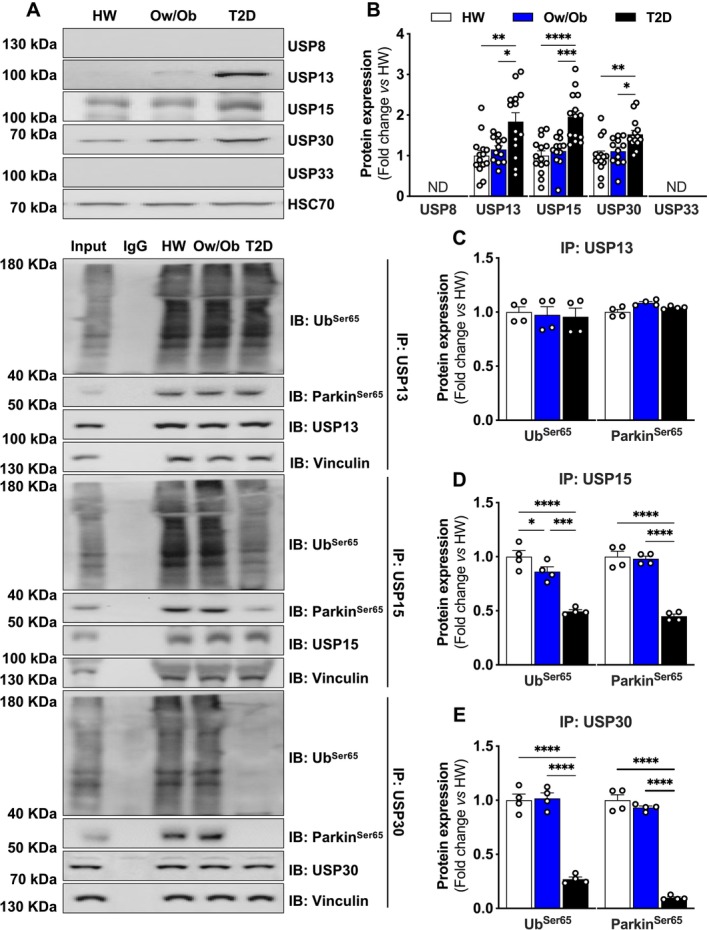
Expression of deubiquitinases and protein–protein interaction in skeletal muscle. (A and B) Representative immunoblots and densitometric quantification of USP8, USP13, USP15, USP30, USP33 and HSC70 (loading control) relative to healthy weight group (*n* = 14 per group). (C–E) Protein–protein interaction between USP13, USP 15 and USP30 with Parkin (*n* = 4 per group). Healthy weight (HW), overweight/obesity (Ow/Ob) and overweight/obesity with type 2 diabetes (T2D). Data are shown as the mean ± SEM. * indicates *p* < 0.05; ** *p* < 0.01; *** *p* < 0.001; **** *p* < 0.0001 for between‐group comparison.

**FIGURE 7:**
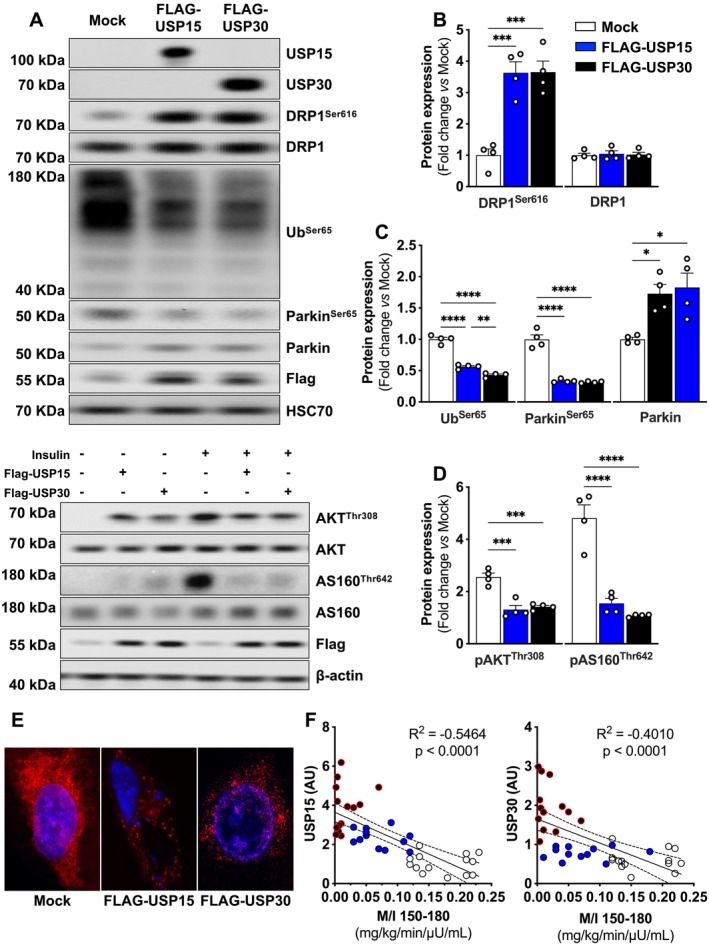
Overexpression of deubiquitinases USP15/30 blunts Ub/Parkin phosphorylation and insulin sensitivity in C2C12 myoblasts. (A–C) Representative immunoblots and densitometric quantification of phosphorylated and total DRP1, ubiquitin phosphorylated, phosphorylated and total Parkin, FLAG and HSC70 (loading control) relative to empty vector (mock) (*n* = 4 biological replicates per group). (A and D) Representative immunoblots and densitometric quantification of phosphorylated and total Akt, phosphorylated and total AS160, FLAG and β‐actin (loading control) ± 30 min of insulin stimulation relative to vehicle (*N* = 4 biological replicates per group). (E) Representative confocal micrographs of C2C12 myoblasts stained with MitoTracker deep red and Hoechst (*n* = 3 biological replicates per group). (F) Relationship between skeletal muscle USP15 and USP30 expression and M/I_150–180_. Data are shown as the mean ± SEM. * indicates *p* < 0.05; ** *p* < 0.01; *** *p* < 0.001; **** *p* < 0.0001 for between‐group comparison.

To explore the mechanisms by which mitochondrial quality control was deficient in the skeletal muscle of T2D, we hypothesized that increased DUB expression may explain impaired Parkin and ubiquitin activation. USPs are an important class of DUB protein that specifically remove covalently linked polypeptides via peptide bonds on the C‐terminal of ubiquitin [21]. To confirm the presence of USPs in human skeletal muscle, we explored the expression of USP8, USP13, USP15, and USP30, which are known to interact with ubiquitin and Parkin [22]. USP8 and USP33 were not detected, whereas USP13, USP15 and USP30 expression were significantly elevated in T2D (Figure 6A,B). We next evaluated protein–protein interactions between USP13, USP15 and USP30 and ubiquitin/Parkin. We found that USP15 and USP30 did interact with ubiquitin and Parkin from the HW and Ow/Ob samples, but not in T2D (Figure 6C–E). To determine whether DUBs directly regulate signalling from the ubiquitin/Parkin axis in muscle, we overexpressed USP15 and USP30 in C2C12 myoblasts. First, we observed that DRP1^Ser616^ phosphorylation was increased in myoblasts overexpressing USP15 and USP30 (Figure 7A,B). Similarly, ubiquitin activation on Ser65 was attenuated by USP15 and to a greater extent in USP30 overexpressing cells, whereas Parkin activation was lower in cells expressing either USP15 and USP30 (Figure 7A,C). Strikingly, cells with USP15 and USP30 overexpression exhibited diminished insulin activation of protein kinase B (Akt) on Threonine 308 and Akt substrate of 160‐kDa protein (AS160) on Threonine 642 phosphorylation sites (Figure 7D). Congruently, USP15 and USP30 overexpressing cells exhibited severely punctate and fragmented mitochondrial networks compared to Mock controls (Figure 7E), and these data were further supported by a negative association with insulin sensitivity in USP15 and USP30 (Figure 7F). Taken together, loss of mitochondrial quality control triggered by insulin resistance, and impaired glucose homeostasis is regulated by DUBs in the skeletal muscle of patients with T2D.

## 4 Discussion

Mitochondrial dynamics is a highly preserved process comprised of cyclic division and reintegration of organelles, which occurs on the outer and inner mitochondrial membranes [7]. As such, maintenance of mitochondrial dynamics is vital to ensuring mitochondrial networks' quality, integrity, and function [9]. We and others have previously described alterations in mitochondrial dynamics in patients with obesity and T2D, but the detailed mechanisms through which this process may contribute to skeletal muscle insulin resistance is still not fully understood [References S2 and S3]. In this study, we investigated differences in mitochondrial dynamics across the human spectrum of insulin resistance and glucose tolerance and assessed the connection between the morphological and functional properties of mitochondria in human skeletal muscle. Herein, we provide the first evidence that (1) DRP1 hyperactivation in T2D is linked to the loss of mitochondrial quality control in the Ub/Parkin axis signalling and (2) USP15/30 elicits the loss of mitochondrial quality control and reduced insulin sensitivity in skeletal muscle. Taken together, these findings highlight a novel mechanism whereby mitochondrial dynamics may regulate insulin sensitivity in the skeletal muscle of T2D.

Increased mitochondrial fragmentation in response to excessive mitochondrial fission has been previously observed in skeletal muscle from patients with obesity and T2D [Reference S3] [23]. These data corroborate our findings that impaired mitochondrial dynamics are associated with reduced insulin sensitivity, characterized by higher FIS1 expression. However, it is well known that DRP1 is the primary driver of mitochondrial fission, causing mitochondrial fragmentation [9, 17], whereas FIS1 or MFF only operate as co‐factors for DRP1 activation [17]. Despite claims that mitochondrial fragmentation is increased in T2D, PINK1 expression, which is responsible for flagging mitochondria following the loss of membrane potential [24], was not different in T2D. It is possible that differences in the patient's phenotype, such as time of T2D diagnosis, age, amount of fat mass, aerobic capacity or medication use, may explain discrepancies between studies.

In mice with diet‐induced obesity and insulin resistance, partial loss of DRP1 was sufficient to enhance skeletal muscle insulin sensitivity [Reference S2]. This result was attributed to reduced mitochondrial reactive oxygen species (mtROS) production, resulting from rebalanced mitochondrial dynamics and integrity. Although our findings are consistent with others showing no defects in mitochondrial respiration in T2D [4, 22], we did not measure mtROS production, and so we cannot entirely rule it out as a potential mediator. However, it is unlikely that mtROS plays a role in controlling DRP1, given that changes in mitochondrial dynamics far precede the rise in hydrogen peroxide emission in insulin‐resistant skeletal muscle [25]. Conversely, studies have revealed that DRP1 interacts directly with phospholipids, such as cardiolipin, to coordinate DRP1 activation and oligomerization [26]. Because cardiolipin is reduced in T2D [1] and other phospholipids are known to play a role in skeletal muscle insulin sensitivity [27], it is plausible that reduced phospholipids in skeletal muscle of T2D could exacerbate DRP1 activation and indirectly increase mtROS [28].

Mitochondrial proteases and chaperones are recruited in response to mtUPR to facilitate degradation of misfolded or damaged proteins that cannot be refolded adequately [29]. We identified a reduction in mtUPR activation as represented by impairments in the COX2/SDH (CII) ratio in Ob and T2D. However, HSP60, HSP90, LonP1 and ClpP, proteins that play a role in refolding and degradation [30], were solely reduced in T2D. Conversely, YME1L expression was only increased in T2D. YME1L is required to maintain mitochondrial morphology, and depletion of YME1L leads to mitochondrial fragmentation [31]. Additionally, COX2, a mitochondrial protein involved in the mtUPR signalling, is a substrate of YME1L that accumulates in the IMM. Incomplete COX2 assembly may lead to an imbalanced protein/lipid ratio in the mitochondrial membrane that changes the membrane fluidity, contributing to the disorganized cristae morphology observed in T2D [31]. Thus, the greater YME1L expression in skeletal muscle in the T2D group may be a compensatory mechanism to counter increased levels of mitochondrial fragmentation.

One of the well‐understood pathways for mitophagy involves the kinases PINK1 and Parkin [32]. PINK1 senses mitochondrial stress through membrane potential, and thus, PINK1 expression is low in healthy mitochondrial networks. PINK1 accumulates on damaged mitochondria, resulting in the phosphorylation of ubiquitin chains and Parkin, leading to autophagy receptor recruitment and autophagosome formation [33]. Interestingly, inactive PINK1 cannot activate or recruit Parkin to mitochondria, indicating that PINK1 kinase activity is required to drive mitophagy [34]. In this regard, PINK1 activates Parkin by directly phosphorylating Serine 65 in the ubiquitin domain of Parkin, as well as an analogous Serine 65 residue on ubiquitin [35]. Our study provides the first evidence that skeletal muscle Parkin and ubiquitin phosphorylation at Serine 65 sites is defective in patients with T2D. This was additionally evidenced by downstream defects of targets related to mitophagy, such as p62 and LC3II, and a reduced number of autophagic vacuoles surrounding mitochondria.

The reduced levels of ubiquitinated proteins observed in the T2D group suggest that elevated DUB activity may serve a compensatory role, reflecting the involvement of DUBs in recycling free ubiquitin to maintain the cellular ubiquitin pool [36, 37]. However, given that free ubiquitin expression was comparable between groups in our study, it is plausible that additional factors contribute to the observed differences in ubiquitinated protein abundance. These factors may include augmented proteasomal degradation, which depletes ubiquitinated proteins while preserving free ubiquitin levels [36], or a redistribution between conjugated and free ubiquitin states without affecting total ubiquitin levels [37]. Future studies should focus on investigating DUB and proteasome activity in T2D skeletal muscle to better understand these dynamics. DUBs have also received extensive attention as potential regulators of mitophagy, more specifically in terms of Parkin's role as a ubiquitin ligase. Out of the ~80 active DUBs identified in the human genome, USP15, USP30 and USP35 have been reported to deubiquitinate substrates of Parkin directly [35]. In that regard, USP15 is a cytosolic DUB that counteracts Parkin‐mediated mitochondrial ubiquitination and mitophagy [21]. USP30, another DUB located on the mitochondrial outer membrane, can directly oppose Parkin's actions [8, 38]. It has been proposed that increasing USP15/30 reverses the ubiquitination of Parkin substrates located on mitochondria, reducing the recruitment of Parkin to damaged mitochondria and impaired mitophagy [8, 21].

The direct impact of DUB proteins on insulin sensitivity in skeletal muscle of T2D remains unclear. Ubiquitination of the protein kinase Akt prior to its phosphorylation is required for its activity, and DUB activity can remove K63‐linked polyubiquitin chains on Akt to restrict PI3K‐Akt signalling in muscle [39]. Indeed, our data demonstrated a reduced expression of K63‐linked polyubiquitin chains in T2D group. DUB‐mediated deubiquitination of Akt reduces phosphorylation at Threonine 308 and Serine 473 sites [39], and both phosphorylation sites play an important role in maintaining muscle insulin sensitivity [40]. Consequently, K63‐linked ubiquitin chains can act as non‐proteolytic signals involved in processes such as glucose uptake [39] and mitophagy [41].This finding suggests that increased DUB activity in T2D skeletal muscle may remove K63‐linked chains, impairing both insulin signalling and mitochondrial quality control. Further investigation is required to confirm this idea.

In conclusion, patients with T2D exhibit loss of mitochondrial content which may be explained by hyperactivation of DRP1. To this end, DUB‐induced Ub/Parkin axis inactivation reduces skeletal muscle insulin sensitivity. Collectively, these findings advance our understanding of how impaired mitochondrial dynamics and quality control may contribute to skeletal muscle insulin resistance and the manifestation of T2D and provides key evidence that DUB antagonists may play an important role in preventing or treating T2D.

## Conflicts of Interest

c.e.F. is currently an investigator at Mission Therapeutics, a company with commercial interest in deubiquinating enzymes (DUB) drug discovery and development. Mission Therapeutics had no role in the design and conduct of the study, collection, analysis or interpretation of the data, manuscript preparation or review.

## Supporting information


**Table S1** Commercially available reagents and resources used in the study.
**Table S2.** Baseline characteristics of patients with a healthy weight (HW), overweight/obesity (Ow/Ob) or overweight/obesity with type 2 diabetes (T2D). Data are mean ± SD or *n* (%). ACE, angiotensin‐converting enzyme; ANG II, angiotensin II; GLP‐1, glucagon‐like peptide 1.
**Figure S1.** Representative unmerged and merged confocal micrographs. Representative confocal micrographs of TMRM (Red), MitoTracker Deep Red (Green), Hoechst (Blue) and resulting merged stacks in HW, Ow/Ob and T2D. Images were acquired at 63× magnification.
**Figure S2.** Between‐group differences in expression of acetylation markers. (A and B) Representative immunoblots and densitometric quantification of global acetylated‐lysine, SIRT1, SIRT3, SIRT5, PGAM5 and HSC70 (loading control) relative to healthy weight group (*n* = 14 per group). (C and D) Protein–protein interaction between DRP1 and acetylated‐lysine (*n* = 4 per group). Healthy weight (HW), overweight/obesity (Ow/Ob) and overweight/obesity with type 2 diabetes (T2D). Data are shown as the mean ± SEM. * indicates *p* < 0.05 for between‐group comparison.
**Figure S3.** Between‐group differences in free ubiquitin and K‐48/K63‐linked polyubiquitination. (A and B) Representative immunoblots and densitometric quantification of free ubiquitin normalized to Ponceau S staining expressed relative to HW (*n* = 14 per group). (A–E) Representative immunoblots and densitometric quantification of K48‐ and K63‐linked polyubiquitination normalized to HSC70 expressed relative to HW (*n* = 14 per group). Data are shown as the mean ± SEM. * indicates *p* < 0.05 for between‐group comparison.

## Data Availability

Data and resources are available from the corresponding author upon reasonable request.
