# Supplementary material for: Deubiquitinating Enzymes Regulate Skeletal Muscle Mitochondrial Quality Control and Insulin Sensitivity in Patients With Type 2 Diabetes
Source: J Cachexia Sarcopenia Muscle. 2025 Mar 4;16(2):e13763. doi: 10.1002/jcsm.13763 (PMC11876994; doi:10.1002/jcsm.13763)
Supplement: Supplementary file 1 — Table S1 Commercially available reagents and resources used in the study. Table S2. Baseline characteristics of patients with a healthy weight (HW), overweight/obesity (Ow/Ob) or overweight/obesity with type 2 diabetes (T2D). Data are mean ± SD or n (%). ACE, angiotensin‐converting enzyme; ANG II, angiotensin II; GLP‐1, glucagon‐like peptide 1. Figure S1. Representative unmerged and merged confocal micrographs. Representative confocal micrographs of TMRM (Red), MitoTracker Deep Red (Green), Hoechst (Blue) and resulting merged stacks in HW, Ow/Ob and T2D. Images were acquired at 63× magnification. Figure S2. Between‐group differences in expression of acetylation markers. (A and B) Representative immunoblots and densitometric quantification of global acetylated‐lysine, SIRT1, SIRT3, SIRT5, PGAM5 and HSC70 (loading control) relative to healthy weight group (n = 14 per group). (C and D) Protein–protein interaction between DRP1 and acetylated‐lysine (n = 4 per group). Healthy weight (HW), overweight/obesity (Ow/Ob) and overweight/obesity with type 2 diabetes (T2D). Data are shown as the mean ± SEM. * indicates p < 0.05 for between‐group comparison. Figure S3. Between‐group differences in free ubiquitin and K‐48/K63‐linked polyubiquitination. (A and B) Representative immunoblots and densitometric quantification of free ubiquitin normalized to Ponceau S staining expressed relative to HW (n = 14 per group). (A–E) Representative immunoblots and densitometric quantification of K48‐ and K63‐linked polyubiquitination normalized to HSC70 expressed relative to HW (n = 14 per group). Data are shown as the mean ± SEM. * indicates p < 0.05 for between‐group comparison. [file JCSM-16-e13763-s001.docx]

**Supplementary Materials**

Deubiquitinating Enzymes Regulate Skeletal Muscle Mitochondrial Quality Control and Insulin Sensitivity in Patients with Type 2 Diabetes

Wagner S. Dantas, Elizabeth C. Heintz, Elizabeth R.M. Zunica, Jacob T. Mey, Melissa L. Erickson, Kathryn P. Belmont, Analisa L. Taylor, Gangarao Davuluri, Hisashi Fujioka, Ciarán E. Fealy, Charles L. Hoppel, Christopher L. Axelrod, John P. Kirwan

**Appendix**

**Supplementary Table 1**. List of reagents and resources used in the study.

**Supplementary Table 2.** Baseline demographic characteristics and medication use.

**Supplementary Figure 1.** Representative unmerged and merged confocal micrographs.

**Supplementary Figure 2.**  Between-group differences in expression of acetylation markers.

**Supplementary Figure 3.** Between-group differences in free ubiquitin and K-48/K63-linked polyubiquitination.

**Supplementary References.** List of supplementary references to the primary manuscript.

**Supplementary Table 1**. Commercially available reagents and resources used in the study.

| **REAGENT or RESOURCE** | **SOURCE** | **IDENTIFIER** |
| --- | --- | --- |
| **Commercial products** | | |
| MitoTracker Deep Red | Invitrogen | Cat# M22426 |
| TMRM | Invitrogen | Cat# I34361 |
| DAPI | Cell Signaling (1:2000) | Cat# 8961 |
| Pierce™ BCA Protein Assay | ThermoFisher Scientific | Cat# 23225 |
| Protein A/G Plus-Agarose | Santa Cruz Biotechnology | Cat# sc-2003 |
| FLAG-USP15 | Addgene | Cat# 22570 |
| FLAG-USP30 | Addgene | Cat# 22578 |
| FLAG-Mock | Addgene | Cat# 17519 |
| **Antibodies** | | |
| DRP1 Ser 616 | Cell Signaling (1:1000) | Cat# 3455 |
| DRP-1 | Cell Signaling (1:2000) | Cat# 8570 |
| MFF Ser 146 | Cell Signaling (1:2000) | Cat# 49281 |
| MFF | Cell Signaling (1:2000) | Cat# 84580 |
| Mid49 | Proteintech (1:2000) | Cat# 20164–1-AP |
| Mid51 | Proteintech (1:2000) | Cat# 20164-1-AP |
| PINK Ser 228 | ThermoFisher Scientific (1:1000) | Cat# PA5-105356 |
| PINK1 Thr 257 | Abcam (1:1000) | Cat# ab303532 |
| PINK1 | Abcam (1:2000) | Cat# ab23707 |
| Parkin Ser 65 | ThermoFisher Scientific (1:1000) | Cat# PA5-114616 |
| Ub Ser 65 | Cell Signaling (1:1000) | Cat# 62802 |
| Parkin | Cell Signaling (1:2000) | Cat# 4211 |
| Polyubiquination | Cell Signaling (1:2000) | Cat# 20326 |
| MFN1 | Proteintech (1:2000) | Cat# 13798-1-AP |
| Akt Thr 308 | Cell Signaling (1:750) | Cat# 4056 |
| Akt | Cell Signaling (1:750) | Cat# 9272 |
| AS160 Thr 642 | Cell Signaling (1:750) | Cat# 8881 |
| AS160 | Cell Signaling (1:750) | Cat# 2670 |
| AMPK Thr 172 | Cell Signaling (1:1000) | Cat# 2531S |
| AMPK | Cell Signaling (1:1000) | Cat# 2532S |
| P70 Thr 389 | Cell Signaling (1:1000) | Cat# 9234S |
| P70 | Cell Signaling (1:1000) | Cat# 2708S |
| MFN2 | Cell Signaling (1:1000) | Cat# 9482 |
| OPA1 | Proteintech (1:2000) | Cat# 27733-1-AP |
| FIS1 | Proteintech (1:2000) | Cat# 10956-1-AP |
| OXPHOS | Abcam (1:2000) | Cat# ab110411 |
| HSP60 | Proteintech (1:2000) | Cat# 15282-1-AP |
| HSP70 | Proteintech (1:2000) | Cat# 25405-1-AP |
| HSP90 | Proteintech (1:2000) | Cat# 13171-1-AP |
| LonP1 | Proteintech (1:2000) | Cat# 15440-1-AP |
| YME1L1 | Proteintech (1:2000) | Cat# 11510-1-AP |
| CLpP | Proteintech (1:2000) | Cat# 15698-1-AP |
| p62 | Proteintech (1:2000) | Cat# 18420–1-AP |
| Beclin1 | Proteintech (1:2000) | Cat# 66665–1-Ig |
| LC3II | Novus Biologicals (1:3000) | Cat# NB910-40752 |
| SIRT1 | Proteintech (1:2000) | Cat# 13161-1-AP |
| SIRT3 | Cell Signaling (1:1000) | Cat# 2627 |
| SIRT5 | Cell Signaling (1:1000) | Cat# 8779 |
| Acetylated-Lysine | Cell Signaling (1:1000) | Cat# 9411 |
| PGC1α | Santa Cruz Biotechnology (1:1000) | Cat# sc-518025 |
| TFAM | Cell Signaling (1:1000) | Cat# 8076 |
| VDAC | Proteintech (1:4000) | Cat# 10866–1-AP |
| USP8 | Proteintech (1:6000) | Cat# 27791-1-AP |
| USP13 | Proteintech (1:4000) | Cat# 16840-1-AP |
| USP15 | Proteintech (1:6000) | Cat# 14354-1-AP |
| USP 15 | Cell Signaling (1:2000) | Cat# 66310 |
| USP30 | Proteintech (1:1000) | Cat# 15402-1-AP |
| USP33 | Proteintech (1:4000) | Cat# 20445-1-AP |
| PGAM5 | Proteintech (1:6000) | Cat# 28445-1-AP |
| HSC70 | Santa Cruz Biotechnology (1:4000) | Cat# 7298 |
| Vinculin | Cell Signaling (1:2000) | Cat# 18799 |
| K63-linkage specific polyubiquitin | Cell Signaling (1:1000) | Cat# 12930S |
| K48-linkage specific polyubiquitin | Cell Siganling (1:1000) | Cat# 4289S |
| Goat anti-rabbit Ig | Millipore (1:8000) | Cat# AP132P |
| Peroxidase Conjugated | Sigma-Aldrich | Cat# P8375 |
| Goat anti-mouse IgG | Millipore (1:8000) | Cat# AP308P |
| (H+L) HRP Conjugated | Cytiva | NA9340 |
| Veriblot IP detection | Abcam (1:8000) | Cat# ab131366 |
| DAPI | Cell Signaling (1:2000) | Cat# 8961 |

**Supplementary Table 2.** Baseline characteristics of patients with a Healthy Weight (HW), Overweight/Obesity (Ow/Ob), or Overweight/Obesity with Type 2 Diabetes (T2D).

|  | **HW**  **(n = 23)** | **Ow/Ob**  **(n = 15)** | **T2D**  **(n = 20)** |
| --- | --- | --- | --- |
| Sex (M/F) | (11/12) | (11/4) | (5/15) |
| **Ethnicity** |  |  |  |
| Black/African Americans | 3 (13.1) | 2 (13.3) | 7 (35.0) |
| Asians | 6 (26.1) | 3 (20.1) | 1 (5.0) |
| Caucasians | 12 (52.2) | 8 (53.3) | 11 (55.0) |
| Hispanics/Latinos | 2 (8.6) | 2 (13.3) | 1 (5.0) |
| **Medication** |  |  |  |
| Antidepressants | 0 (0.0) | 1 (6.6) | 6 (30.0) |
| β-blockers | 0 (0.0) | 0 (0.0) | 0 (0.0) |
| ACE inhibitors | 0 (0.0) | 0 (0.0) | 4 (20.0) |
| Calcium channel blockers | 0 (0.0) | 0 (0.0) | 5 (25.0) |
| ANG II receptor antagonists | 0 (0.0) | 0 (0.0) | 2 (10.0) |
| Diuretics | 0 (0.0) | 0 (0.0) | 4 (20.0) |
| Metformin | 0 (0.0) | 0 (0.0) | 10 (50.0) |
| Insulin | 0 (0.0) | 0 (0.0) | 0 (0.0) |
| GLP-1 agonists | 0 (0.0) | 0 (0.0) | 5 (25.0) |
| Sulfonylureas | 0 (0.0) | 0 (0.0) | 2 (10.0) |
| SGLT2 inhibitors | 0 (0.0) | 0 (0.0) | 3 (15.0) |
| DPP-4 inhibitors | 0 (0.0) | 0 (0.0) | 2 (10.0) |
| Statins | 0 (0.0) | 0 (0.0) | 8 (40.0) |
| Salicylates | 0 (0.0) | 0 (0.0) | 4 (20.0) |

Data are mean ± SD or n (%). ACE, angiotensin-converting enzyme; ANG II, angiotensin II; GLP-1, glucagon-like peptide 1.

**Supplementary Figure 1.** Representative unmerged and merged confocal micrographs.

Representative confocal micrographs of TMRM (Red), Mitotracker Deep Red (Green), Hoechst (Blue), and resulting merged stacks in HW, Ow/Ob, and T2D. Images were acquired at 63X magnification.

**Supplementary Figure 2.**  Between-group differences in expression of acetylation markers.

(A and B) Representative immunoblots and densitometric quantification of global acetylated-lysine, SIRT1, SIRT3, SIRT5, PGAM5, and HSC70 (loading control) relative to healthy weight group (n = 14 per group). (C and D) Protein-protein interaction between DRP1 and acetylated-lysine (n = 4 per group). Healthy Weight (HW), Overweight/Obesity (Ow/Ob), and Overweight/Obesity with Type 2 Diabetes (T2D). Data are shown as the mean ± SEM. *indicates *p*<0.05 for between-group comparison.

**Supplementary Figure 3**. Between-group differences in free ubiquitin and K-48/K63-linked polyubiquitination.

(A and B) Representative immunoblots and densitometric quantification of free ubiquitin normalized to Ponceau S. staining expressed relative to HW (n = 14 per group). (A-E) Representative immunoblots and densitometric quantification of K48- and K63-linked polyubiquitination normalized to HSC70 expressed relative to HW (n = 14 per group). Data are shown as the mean ± SEM. *indicates *p*<0.05 for between-group comparison.

**Supplementary References**

SR1. Detmer SA, and Chan DC. Functions and dysfunctions of mitochondrial dynamics. *Nat Rev Mol Cell Biol.* 2007;8(11):870-9.

SR2. Kugler BA, Lourie J, Berger N, Lin N, Nguyen P, DosSantos E, et al. Partial skeletal muscle-specific Drp1 knockout enhances insulin sensitivity in diet-induced obese mice, but not in lean mice. Mol Metab. 2023;77:101802.

SR3. Houzelle A, Jorgensen JA, Schaart G, Daemen S, van Polanen N, Fealy CE, et al. Human skeletal muscle mitochondrial dynamics in relation to oxidative capacity and insulin sensitivity. Diabetologia. 2021;64(2):424-36.

SR4. Zorzano A, Liesa M, and Palacin M. Role of mitochondrial dynamics proteins in the pathophysiology of obesity and type 2 diabetes. Int J Biochem Cell Biol. 2009;41(10):1846-54.

SR5. Rovira-Llopis S, Banuls C, Diaz-Morales N, Hernandez-Mijares A, Rocha M, and Victor VM. Mitochondrial dynamics in type 2 diabetes: Pathophysiological implications. Redox Biol. 2017;11:637-45.

SR6. Solomon TP, Haus JM, Kelly KR, Cook MD, Filion J, Rocco M, et al. A low-glycemic index diet combined with exercise reduces insulin resistance, postprandial hyperinsulinemia, and glucose-dependent insulinotropic polypeptide responses in obese, prediabetic humans. Am J Clin Nutr. 2010;92(6):1359-68.

SR7. Steele R, Wall JS, De Bodo RC, and Altszuler N. Measurement of size and turnover rate of body glucose pool by the isotope dilution method. *Am J Physiol.* 1956;187(1):15-24.

SR8. Evans WJ, Phinney SD, and Young VR. Suction applied to a muscle biopsy maximizes sample size. *Med Sci Sports Exerc.* 1982;14(1):101-2.

SR9. Ko F, Abadir P, Marx R, Westbrook R, Cooke C, Yang H, et al. Impaired mitochondrial degradation by autophagy in the skeletal muscle of the aged female interleukin 10 null mouse. Exp Gerontol. 2016;73:23-7.

SR10. Eskelinen EL. To be or not to be? Examples of incorrect identification of autophagic compartments in conventional transmission electron microscopy of mammalian cells. Autophagy. 2008;4(2):257-60.

SR11. Yla-Anttila P, Vihinen H, Jokitalo E, and Eskelinen EL. Monitoring autophagy by electron microscopy in Mammalian cells. Methods Enzymol. 2009;452:143-64.
